# Effect of *Quamoclit angulata* Extract Supplementation on Oxidative Stress and Inflammation on Hyperglycemia-Induced Renal Damage in Type 2 Diabetic Mice

**DOI:** 10.3390/antiox9060459

**Published:** 2020-05-27

**Authors:** Ji Eun Park, Heaji Lee, Hyunkyung Rho, Seong Min Hong, Sun Yeou Kim, Yunsook Lim

**Affiliations:** 1 Department of Food and Nutrition, Kyung Hee Univerity, 26 Kyung Hee-Daero, Dongdamun-Gu, Seoul 02447, Korea; gh1003@khu.ac.kr (J.E.P.); ji3743@khu.ac.kr (H.L.); rho0408@khu.ac.kr (H.R.); 2 Department of Pharmacognosy, College of Pharmacy, Gachon University, Incheon 21936, Korea; hongsm0517@gmail.com (S.M.H.); sunnykim@gachon.ac.kr (S.Y.K.)

**Keywords:** *Quamoclit angulata*, type 2 diabetes, kidney damage, inflammation, oxidative stress, apoptosis, fibrosis

## Abstract

Type 2 diabetes mellitus (T2DM) is caused by abnormalities of controlling blood glucose and insulin homeostasis. Especially, hyperglycemia causes hyper-inflammation through activation of NLRP3 inflammasome, which can lead to cell apoptosis, hypertrophy, and fibrosis. *Quamoclit angulata* (QA), one of the annual winders, has been shown ameliorative effects on diabetes. The current study investigated whether the QA extract (QAE) attenuated hyperglycemia-induced renal inflammation related to NLRP inflammasome and oxidative stress in high fat diet (HFD)-induced diabetic mice. After T2DM was induced, the mice were treated with QAE (5 or 10 mg/kg/day) by gavage for 12 weeks. The QAE supplementation reduced homeostasis model assessment insulin resistance (HOMA-IR), kidney malfunction, and glomerular hypertrophy in T2DM. Moreover, the QAE treatment significantly attenuated renal NLRP3 inflammasome dependent hyper-inflammation and consequential renal damage caused by oxidative stress, apoptosis, and fibrosis in T2DM. Furthermore, QAE normalized aberrant energy metabolism (downregulation of p-AMPK, sirtuin (SIRT)-1, and PPARγ-coactivator α (PGC-1 α)) in T2DM mice. Taken together, the results suggested that QAE as a natural product has ameliorative effects on renal damage by regulation of oxidative stress and inflammation in T2DM.

## 1. Introduction

Diabetes mellitus (DM) is considered as a metabolic disease that results in impaired glucose and insulin homeostasis [1]. Especially, insulin resistance caused by hyperglycemia, is the worldwide epidemic that is accompanied by various complications in type 2 DM (T2DM) [2]. The early stage of nephropathy (DN) is characterized by the structural changes of kidney such as damage of the glomerular basement membrane (GBM), enlargement of the mesangial cells, glomerulosclerosis, and fibrosis and renal function failure including microalbuminuria and reduced glomerular filtration rate (GFR) [3,4].

The main cause of renal damage is the hyperglycemic condition in T2DM. Hyperglycemia leads to overproduction of reactive oxygen species (ROS), which potentially causes oxidative stress and activates various cytokines, chemokines, and growth factors. Oxidative stress results from an imbalance between oxidants and antioxidants such as NAD(P)H quinone dehydrogenase-1 (NQO1), hemeoxygenase-1 (HO-1), catalase, superoxide dismutase (SOD), and glutathione peroxidase (GPx) [5,6,7]. The pathogenic changes caused by hyperglycemia-induced oxidative stress modify normal cell signaling and induce hyper-inflammation, apoptosis [8]. Moreover, formation of advanced glycation end products (AGEs) and activation of the AGE receptor due to hyperglycemia directly promote inflammatory states in the kidney by increasing oxidative stress [9].

Furthermore, oxidative stress activates the nucleotide-binding oligomerization domain (NOD)-like pyrin domain containing receptor 3 (NLRP3) inflammasome [10,11]. Activated NLRP3 recruits the apoptosis-associated speck-like protein containing a caspase recruitment domain (ASC) and pro-caspase-1, and contributes to the maturation of interleukin-1β (IL-1β) by activating caspase-1. NLRP3 inflammasome and mature IL-1β activate a transcription factor, nuclear factor-κB (NF-κB), and enhance multiple proinflammatory factors such as tumor necrosis factor-α (TNF-α), interleukin-6 (IL-6), and inducible nitric oxide synthase (iNOS) [12]. Hence, the activation of NLRP3 inflammasome contributes to chronic inflammatory response as well as insulin resistance in diabetes [13,14].

Moreover, chronic hyperglycemia-induced oxidative stress and hyper-inflammation accelerate renal apoptosis [5,13,15]. Cellular apoptosis is regulated by caspases through an extrinsic and intrinsic pathway. In ongoing-diabetes, hyperglycemia induces ROS-related apoptosis by increasing the Bax/Bcl-2 ratio, which is associated with progressive activation of pro-apoptotic caspase-3 [15,16]. ROS also increases protein kinase C (PKC), which activates transcription of transforming growth factor-β (TGF-β) [17]. In general, TGF-β leads to proliferation of fibroblasts and activates α-smooth muscle actin (α-SMA), which accommodates collagen formation and cellular hypertrophy. TGF-β also causes the proliferation of mesangial cells, consequentially leading to kidney fibrosis [18]. Therefore, suppression of renal cell apoptosis and pro-fibrotic change along with NLRP3 inflammasome related hyper-inflammation would be an effective target strategy on alleviating renal damage and the progress to DN [13].

Furthermore, abnormal energy metabolism in the kidney can cause renal damage. Sirtuin1 (SIRT1) mediates inflammatory signaling and apoptosis as a molecular response to glucotoxicity via deacetylation and inhibition of transcription factor NF-κB [19]. When the AMP/ATP ratio increases, 5′ adenosine monophosphate-activated protein kinase (AMPK) alleviates renal inflammation causing aberrant energy accumulation. The SIRT1/AMPK signaling pathway along with the peroxisome proliferator-activated receptor γ-coactivator α (PGC-1α) also suppresses renal hyper-inflammation and oxidative stress [20]. Hence, amelioration of SIRT1/AMPK signaling would be a possible therapeutic approach for diabetic renal damage.

In recent years, many medicinal plants have been reported as antidiabetic natural products including banaba, fenugreek, gymnema, yerba mate, etc. Among these plants, *Quamoclit angulata* (QA) is emerging as a source of therapeutic substance for diabetes and its complications. Although QA has not been fully investigated, a previously reported patent has shown that herbal agents of the *Quamoclit angulata* extract (QAE) decreased the fasting blood glucose (FBG) level and hemoglobin A1c (HbA1c) production by stimulating insulin secretion in pancreatic β cell. QAE also attenuated albuminuria, which is a major factor of DN in diabetic mice. Furthermore, QAE ameliorated angiogenesis by reducing the mRNA level of vascular endothelial growth factor (VEGF) in the ARPE 19 cell [21]. Nevertheless, little research has investigated the effects of QAE supplementation on renal damage in a hyperglycemic condition by molecular mechanisms. Hence, we examined if QAE supplementation has protective roles in renal damage via modulation of oxidative stress and inflammation in high fat diet-induced diabetic mice.

## 2. Materials and Methods

### 2.1. Quamoclit Angulata Extracts (QAE)

QA was obtained at Jeju, Korea. Aerial parts without the seed of QA (50 g) were extracted with 400 mL of water by incubation at 50 °C for 1 h. The sticky solid extract (50 g) was suspended in water, added to 1 kg of activated charcoal at room temperature for 1.5 h. After incubation, water, 20% ethanol fraction, and charcoal were removed through centrifugation and filtration (0.45 μm). Fractions were mixed, concentrated in vacuo, and frozen to dry. The yields of hot water extract and activated charcoal fractions were 25% and 11%, respectively.

### 2.2. Identification of Candidate Compounds of QAE

The standardization of QA was analyzed by using the HPLC system (Waters Corp., Milford, MA, USA) consisting of a separation module (e2695) and a photodiode array (PDA) detector. Twenty milligrams of dried QA were dissolved in 50% methanol/water. Protocatechuic acid, chlorogenic acid, syringic acid, myricetin, and quercetin were used as standard compounds and dissolved in methanol. For the analysis of each compound or sample, a Kromasil C^18^ column (150 × 4.6 mm, 5 µm) was used and a column temperature was set at 30 °C. The mobile phase consists of 3% acetic acid/water (solvent A) and methanol (solvent B) using a gradient program of 0–10% (B) in 0–10 min, 10–70% (B) in 10–44 min, 70–100% (B) in 44–50 min. The calibration was linear in a range of 0.1–1000 µg/mL for these five compounds. The flow rate was 0.9–1.0 mL/min and the PDA detector was set at 280 nm for acquiring chromatograms.

### 2.3. Animals Experiments

Male C57BL/6 mice at five weeks were housed in two or three per cages and maintained in a constant environment (temperature (22 ± 1 °C), humidity (50 ± 5%), and 12 h light/12 h dark cycle). After seven days of adaptation, the mice were randomly allocated into two groups. The first group was a non-diabetic control group (NC), which was fed an AIN-93G diet (10% kcal fat, Research Diets, New Brunswick, NJ, USA). The second was a diabetic group (DM), which was fed a high fat diet (40% kcal fat, Research Diets, New Brunswick, NJ, USA) for four weeks.

Then, the diabetic group received an intraperitoneal administration of 30 mg/kg body weight (BW) of streptozotocin (Sigma-Aldrich, St. Louis, MO, USA) in a citric acid buffer (pH 4.4). The NC mice received an equivalent amount of solvent. After five weeks from the last injection, fasting blood glucose (FBG) levels were measured once per week during the whole period of the animal experiment. Mice with FBG >140.4 mg/dL (7.8 mmol/L) more than two times were considered as the diabetic condition. The diabetes induction protocol was referred to the previous study by Zhang et al. [22].

Mice were separated in four groups; (1) CON: Non-diabetic normal mice were gavaged with distilled water, (2) DMC: Diabetic mice were gavaged with distilled water, (3) LQ: Diabetic mice were gavaged with a low dosage of QAE (5 mg/kg/day), (4) HQ: Diabetic mice were gavaged with a high dosage of QAE (10 mg/kg/day). QAE was dissolved in distilled water. Body weight, food intake, and fasting blood glucose level were weekly monitored during the animal experiment.

The animals were sacrificed after 12 weeks of oral supplementation. Blood was collected in a heparin (Sigma-Aldrich, St. Louis, MO, USA) coated syringe from the heart, centrifuged at 850 g at 4 °C for 10 min to obtain plasma. The kidney was removed from mice and stored at −80 °C before the experiment. All experiments with mice were approved by the Institutional Animal Care and Use Committee of Kyung Hee University (KHUASP(SE)-16-005 on 14 June, 2019).

### 2.4. Hemoglobin A1c (HbA1c) and Plasma Insulin Assay

HbA1c levels were measured using enzyme-linked immunosorbent assay (ELISA) commercial kits (Crystal Chem., Downers Grove, Elk Grove Village, IL, USA) according to directions of the manufacturer within two weeks from the sample collection.

The plasma insulin level was measured using ELISA kits (RayBiotech, Inc., Norcross, GA, USA). The homeostasis model assessment of insulin resistance (HOMA-IR) values were calculated as follows:

HOMA-IR = fasting insulin (mmol/L) × fasting glucose (μU/mL)/22.5

### 2.5. Oral Glucose Tolerance Test (OGTT)

Fasted mice were administrated for 16 h with a 50% glucose solution (2 g/kg). The blood glucose level was detected at 0, 15, 30, 60, 90, and 120 min using a glucometer (OneTouch, LifeScan Inc., Malvern, PA, USA). The area under the curve (AUC) values of OGTT are calculated according to the trapezoidal rule as follows:

AUC = ∑ (((blood glucose) _i_ + (blood glucose) _i−1_) × ((time) _i_ – (time) _i−1_)/2) (i = time sequence)

### 2.6. Renal Function Test

Urine samples were collected during three phases of the experiment (0–4 weeks; initial, 4–8 weeks; mid, and 8–12; late-points). Urinary albumin excretion was determined by the albumin assay kit (Bioassay, Hayward, CA, USA). The concentrations of urinary and plasma creatinine were calculated from interpolating the results of optical density at 515 nm into a standard curve. Concentrations of BUN were measured in accordance with the manufacturer’s instructions using a commercial kit (Asan pharmaceutical, Seoul, South Korea).

### 2.7. Histological Observation of Kidney

Kidney tissues were fixed in 10% formaldehyde and then dehydrated through a series of alcohol. The tissues were cleared in xylene and embedded in paraffin. The sections were cut with a microtome into 5 μm, and stained with hematoxylin and eosin (H&E). Kidney morphology in stained tissue was observed using an optical microscope (Nikon ECLIPSE Ci, Nikon Instrument, Tokyo, Japan).

To calculate the glomerular area in H&E-staining, paraffin-embedded sections were measured by the Canvas 11 software (Deneba, Miami, FL, USA). The Glomerulus area was expressed as the mean of thirty glomeruli per each sample and a minimum of four samples from each group were examined. Area values are reported in μm^2^ × 10^−3^.

### 2.8. Protein Extraction and Western Blot Analysis

The kidneys were ground and lysed on ice for 30 min. The lysate was centrifuged to remove tissue debris at 1945× *g* at 4 °C for 10 min. Each supernatant was centrifuged again at 9078× *g* at 4 °C for 30 min. Then, the final supernatant was collected for cytosolic extract. The pellet was re-crushed in a hypertonic lysis buffer for 1 h, and then the lysate was centrifuged at 9078× *g* at 4 °C for 20 min and the supernatant was used for nuclear extract. The protein concentration was quantified according to a BCA protein assay (ThermoFisher Scientific, Grand Island, NY, USA).

Thirty μg of each protein sample were loaded into an SDS-PAGE and transferred to poly-vinylidine fluoride (PVDF) membranes (Millipore, Marlborough, MA, USA). We used 8~12% SDS-PAGE gel according to the molecular weight (MW) of target protein(s). After the transfer, the membrane was blocked in 1~3% bovine serum albumin (BSA) in a phosphate buffed saline −0.1% Tween 20 (PBS-T), the membrane was incubated at 4 °C with each primary antibody. To detect primary antibodies, respective horseradish peroxide (HRP)-conjugated secondary antibodies were given to membranes. Protein bands were visualized using a chemiluminescent detector (Syngene, Cambridge, UK). Levels of targeted proteins were calculated using Syngene GeneSnap (Syngene, Cambridge, UK).

### 2.9. Statistical Analysis

Data were expressed as mean ± standard error of the mean (SEM). The significant differences between sample groups were determined using one-way ANOVA (significant level = 0.05).

## 3. Results

### 3.1. Identification of Major Natural Compounds in QAE

In this study, five compounds such as protocatechuic acid, chlorogenic acid, syringic acid, myricetin, and quercetin were analyzed by using the HPLC system. As shown in Table 1, the QA extract mainly involved protocatechuic acid (198.86 ± 2.26 µg/g). Meanwhile, chlorogenic acid (54.11 ± 1.81 µg/g), syringic acid (56.38 ± 0.57 µg/g), myricetin (12.42 ± 0.09 µg/g), and quercetin (13.41 ± 0.08 µg/g) were detected in QAE.

### 3.2. Effect of QAE Supplementation on Body Weight, Food Intake, and Kidney Weight in T2DM Mice

Body weight and food intake of all T2DM groups (the DMC group, the LQ group, and the HQ group) were significantly increased compared to the NC group. QAE supplementation for 12 weeks had no effect on body weight change in T2DM mice. With a slightly different result, kidney weight in the DMC group was significantly increased compared to the NC group, and there was no significant difference in the LQ group and the HQ group compared to the DMC group (Table 2).

### 3.3. Effect of QAE Supplementation on Fasting Blood Glucose and Plasma Insulin Levels, Homeostasis Model Assessment of Insulin Resistance (HOMA-IR), and Hemoglobin A1c (HbA1c) in T2DM Mice

Fasting blood glucose level, plasma insulin level, HOMA-IR, and HbA1c level were as follows (Table 3). At the end of the QAE treatment period, there was no difference in the fasting blood glucose level among all groups. The plasma insulin level in the HQ group was significantly lower than that in the DMC group. HOMA-IR was significantly reduced in both QAE treated diabetic groups. The HbA1c level was significantly decreased only in the HQ group compared with the DMC group (Table 3).

### 3.4. Effect of QAE Supplementation on Glucose Homeostasis in T2DM Mice

**Figure 1:**
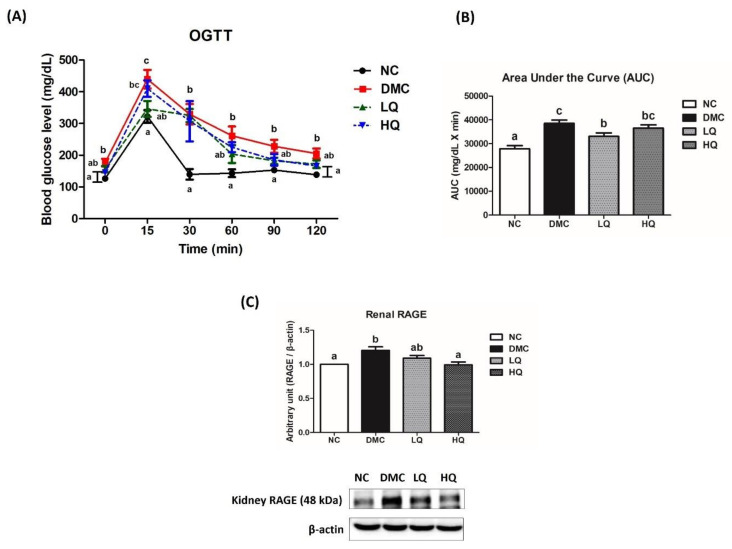
Effect of QAE on (**A**) glucose tolerance, (**B**) glucose area under the curve (AUC), and (**C**) renal receptor of AGE (RAGE) in T2DM mice. Data were expressed as means ± SEM. Mean values with the same superscript letter are not significantly different (*p* < 0.05). NC: Normal mice; DMC: Type 2 diabetic mice; LQ: Type 2 diabetic mice supplemented with a low dose (5 mg/kg/day) of QAE; HQ: Type 2 diabetic mice supplemented with a high dose (10 mg/kg/day) of QAE.

OGTT was performed to estimate insulin resistance and failure of glucose metabolism (Figure 1A), and glucose AUC was calculated as shown in Figure 1B. The figure showed that the DMC group had a high blood glucose level during 120 min after glucose administration compared to the NC group and there was no significant difference in the blood glucose level at 90 min after glucose administration among the DMC group and the QAE treatment groups. The blood glucose level of the LQ group at 120 min was remarkably reduced as compared to the DMC group and glucose AUC of the LQ group was significantly lower than that of the DMC group. On the other hand, the protein level of receptor for advanced glycation end products (RAGE) was remarkably increased in the DMC group compared to that of the NC group (Figure 1C). The HQ group showed a significant reduction of RAGE expression in comparison to the DMC group.

### 3.5. Effect of QAE Supplementation on Kidney Function in T2DM Mice

**Figure 2:**
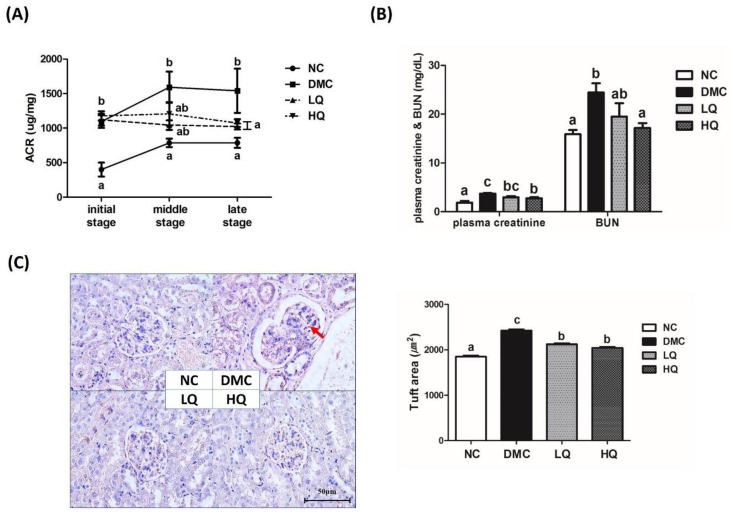
Effect of QAE on (**A**) urine albumin/creatinine ratio (ACR), (**B**) level of plasma creatinine and blood urea nitrogen (BUN), and (**C**) renal morphology and glomerular size in T2DM. Data were expressed as means ± SEM. Mean values with the same superscript letter (a,b and c) are not significantly different (*p* < 0.05). NC: Normal mice; DMC: Type 2 diabetic mice; LQ: Type 2 diabetic mice supplemented with a low dose (5 mg/kg/day) of QAE; HQ: Type 2 diabetic mice supplemented with a high dose (10 mg/kg/day) of QAE.

The ACRs of all T2DM groups were significantly higher than that in the NC group during the entire experimental period (Figure 2A). The ACRs of the QAE treatment groups decreased during the treatment period and showed a significant difference at the late stage of treatment in the diabetic mice. As shown in Figure 2B, the supplementation with a high dose of QAE significantly decreased the plasma creatinine and BUN compared with the DMC group. In representative H&E staining of the kidney (Figure 2C), the DMC group showed glomerular hypertrophy as compared to the NC group, while both the QAE treated groups ameliorated glomerular hypertrophy. The red arrow indicated mesangial expansion in the DMC group compared to the NC group. In the NC group, Bowman’s space was observed as a thin white line. However, Bowman’s space was broadened in the DMC group compared to that in the NC group, and was narrower in the QAE treatment groups than that in the DMC group. In addition, glomerular surface areas in histological sections of renal cortex were quantified to measure the degree of glomerular hypertrophy (Figure 2C). The glomerulus of the DMC group was significantly expanded compared with that of the NC group, while both QAE supplementation groups regardless of dose showed significantly reduced glomerular hypertrophy.

### 3.6. Effect of QAE Supplementation on Oxidative Stress in T2DM Mice

**Figure 3:**
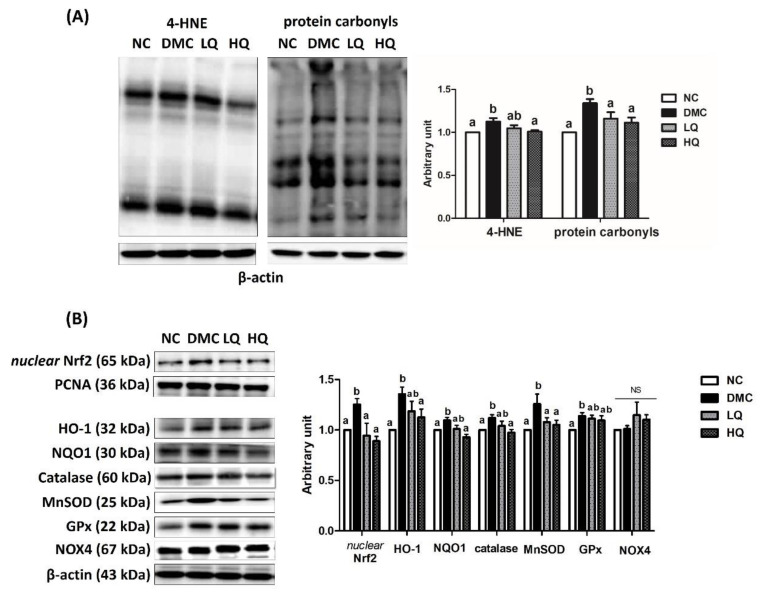
Effect of QAE on renal (**A**) 4-hydroxynonenal (4-HNE) and protein carbonyls and (**B**) antioxidant defense system in T2DM mice. Data were expressed as means ± SEM. Mean values with the same superscript letter (a,b) are not significantly different (*p* < 0.05). NC: Normal mice; DMC: Type 2 diabetic mice; LQ: Type 2 diabetic mice supplemented with a low dose (5 mg/kg/day) of QAE; HQ: Type 2 diabetic mice supplemented with a high dose (10 mg/kg/day) of QAE.

The renal 4-hydroxynonenal (4-HNE) level was examined for assessing lipid peroxidation and the level of renal protein carbonyls was used as a marker of protein oxidation caused by oxidative stress (Figure 3A). In the kidney, the 4-HNE protein level in the DMC group was significantly higher than that in the NC group. The HQ group presented a significant reduction of 4-HNE level compared to that of the DMC group. Renal levels of protein carbonyls in both QAE groups were significantly lower than that in the DMC group. In the DMC group, the protein levels of nuclear Nrf2 and its related markers such as HO-1, NQO1, catalase, MnSOD, and GPx were remarkably higher than those in the NC group. However, both QAE treatments significantly reduced the protein levels of nuclear Nrf2 and MnSOD. Moreover, a high dose of the QAE treatment significantly decreased the protein levels of HO-1, NQO1, and catalase in the diabetic mice. The protein levels of GPx and NOX4 were not significantly different among the DMC group and the QAE treatment groups (Figure 3B).

### 3.7. Effect of QAE Supplementation on Inflammation in T2DM Mice

**Figure 4:**
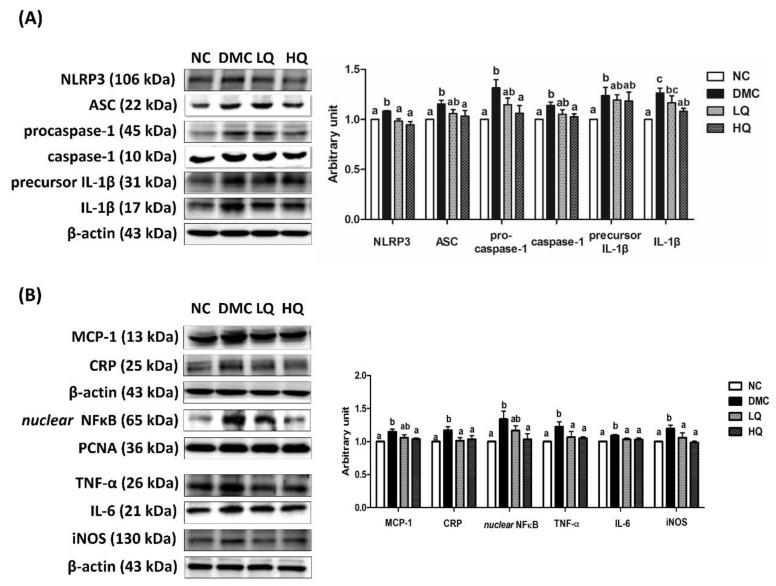
Effect of QAE on renal NLRP3 Inflammasome related hyper-inflammation in T2DM mice. Protein levels of (**A**) nucleotide-binding oligomerization domain-like pyrin domain containing receptor 3 (NLRP3) inflammasome: nucleotide-binding oligomerization domain-like pyrin domain containing receptor 3 (NLRP-3); apoptosis-associated speck-like proteins including caspase recruitment domain (ASC), caspase-1, and interleukin (IL)-1β; and (**B**) markers of pro-inflammatory response: monocyte chemoattractant protein-1 (MCP-1), C-reactive protein (CRP); and nuclear factor kappa B (NF-κB)-related inflammatory response: nuclear factor kappa B (NF-κB), tumor necrosis factor-α (TNF-α), interleukin (IL)-6, and inducible nitric oxide synthase (iNOS). Data were expressed as means ± SEM. Mean values with the same superscript letter (a,b and c) are not significantly different (*p* < 0.05). NC: Normal mice; DMC: Type 2 diabetic mice; LQ: Type 2 diabetic mice supplemented with a low dose (5 mg/kg/day) of QAE; HQ: Type 2 diabetic mice supplemented with a high dose (10 mg/kg/day) of QAE.

The protein level of NLRP3 inflammasome was elevated in the DMC group compared to that of the NC group. However, the level of NLRP3 was significantly decreased in the QAE treatment groups compared to those in the DMC group. However, only a high dose of QAE treatment significantly lowered the protein levels of ASC, procaspase-1, caspase-1, and mature IL-1β in the diabetic mice. The protein levels of precursor IL-1β were not normalized in the QAE treatment groups compared to that in the DMC group (Figure 4A).

Furthermore, the DMC group demonstrated higher levels of inflammation related protein including monocyte chemoattractant protein (MCP)-1, CRP, nuclear NF-κB, TNF-α, IL-6, and iNOS than the NC group (Figure 4B). However, a high dose of QAE treatment in the diabetic mice reversed the protein levels of MCP-1 and nuclear NF-κB to the levels of the NC mice. In addition, the QAE treatment regardless of dose suppressed other inflammatory markers such as CRP, TNF-α, IL-6, and iNOS in the diabetic mice.

### 3.8. Effect of QAE Supplementation on Energy Metabolism in T2DM Mice

**Figure 5:**
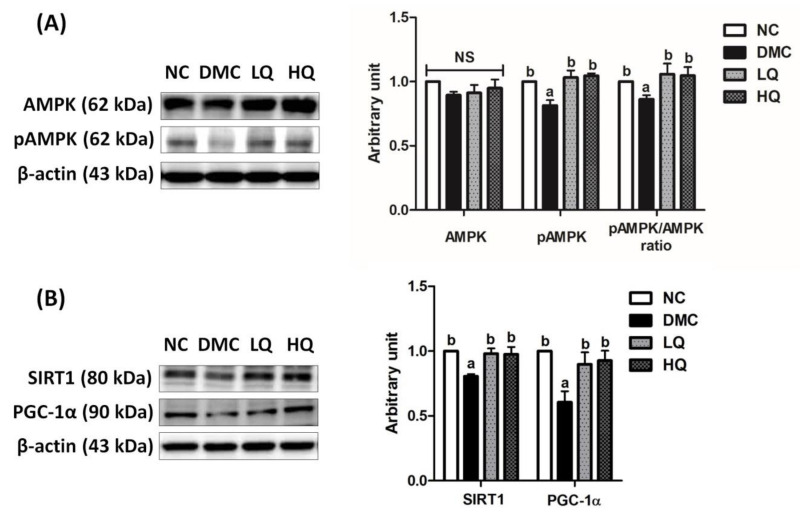
Effect of QAE on renal AMPK/SIRT1 signaling in T2DM mice. (**A**) Phosphorylation of AMPK and (**B**) SIRT-1 and PGC-1α. Data were expressed as means ± SEM. Mean values with the same superscript letter (a,b) are not significantly different (*p* < 0.05). NC: Normal mice; DMC: Type 2 diabetic mice; LQ: Type 2 diabetic mice supplemented with a low dose (5 mg/kg/day) of QAE; HQ: Type 2 diabetic mice supplemented with a high dose (10 mg/kg/day) of QAE.

The protein levels of AMPK were not significantly different among the groups. The protein level of phosphorylated AMPK in the DMC group was significantly declined compared to that of the NC group, but those in the QAE treatment groups were increased compared to the DMC group. In addition, the QAE treatment elevated the pAMPK/AMPK ratio as much as the level of the NC group (Figure 5A). Furthermore, the protein levels of SIRT1 and PGC-1α were significantly declined in the DMC group compared to those in the NC group, but were increased in the QAE treatment groups regardless of dosage (Figure 5B).

### 3.9. Effect of QAE Supplementation on Apoptosis and Fibrosis in T2DM Mice

**Figure 6:**
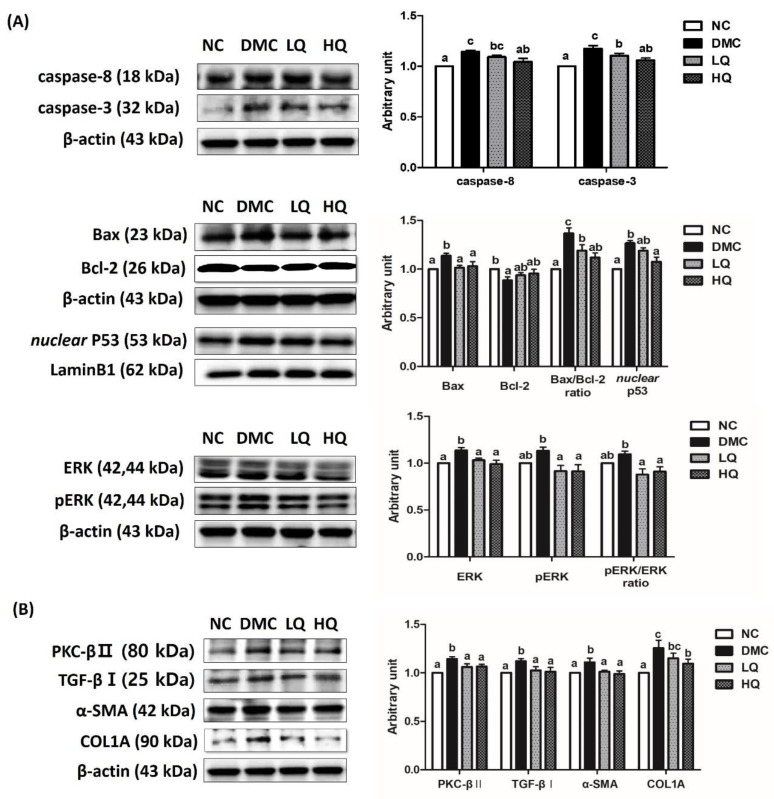
Effect of QAE on renal (**A**) apoptosis and (**B**) fibrosis in T2DM mice. Data were expressed as means ± SEM. Mean values with the same superscript letter (a,b and c) are not significantly different (*p* < 0.05). NC: Normal mice; DMC: Type 2 diabetic mice; LQ: Type 2 diabetic mice supplemented with a low dose (5 mg/kg/day) of QAE; HQ: Type 2 diabetic mice supplemented with a high dose (10 mg/kg/day) of QAE.

The protein levels of caspase-8, caspase-3, and nuclear p53 in the DMC group were significantly higher than those in the NC group, but the high dose of QAE supplementation decreased the protein levels of caspase-8 and p53 than those in the DMC group. Furthermore, the QAE treatment regardless of dose reduced caspase-3 compared to the DMC group (Figure 6A). The protein levels of Bax in the QAE treatment groups were significantly decreased in comparison to that of the DMC group. The QAE treatment regardless of dose remarkably lowered the protein level of Bax/Bcl-2 ratio in the diabetic mice (Figure 6A). In addition, the QAE treatments reduced the protein level of ERK compared to that of the DMC group. At the same time, the protein levels of phosphorylated ERK in the QAE treatment groups were reduced compared to that in the DMC group. Moreover, the pERK/ERK ratio, an index of ERK phosphorylation, in the QAE treatment groups was also decreased compared to that in the DMC group (Figure 6A).

To examine the effect of the QAE supplementation on renal fibrosis, the protein levels of PKC-βII, TGF-β, α-SMA, and COL1A were measured (Figure 6B). The renal protein levels of PKC, TGF-β, α-SMA, and COL1A in the DMC group were significantly higher than those in the NC group. However, the QAE treatments decreased the protein levels of PKC, TGF-β, and α-SMA in comparison to the DMC group, and, in particular, a high dose of the QAE treatment declined the protein level of COL1A in the diabetic mice.

## 4. Discussion

Various studies noticed that many medicinal plants and natural products have potential biological activities. Among these plants, QA is a species of ipomoea morning glory and cultivated as an ornamental plant throughout the tropics. In this study, we aimed to investigate that dietary QAE supplementation could have beneficial effects on NLRP3 inflammasome dependent hyper-inflammation and consequential renal damage by stimulation of AMPK-SIRT1 signaling in type 2 diabetes.

The current study suggested a hypoglycemic effect of QAE presented by decreased plasma insulin, HOMA-IR, and HbA1c. HbA1c is considered as an index of average blood glucose control level, because the HbA1c level tends to increase with the averaged blood glucose levels over preceding three months. A previous study also showed a strong correlation between HbA1c and 6-h fasting glucose levels than overnight FBG levels in diabetic mice [23]. In this study, the QA supplementation ameliorated plasma insulin level, HOMA-IR, and HbA1c compared to the DMC group, although did not significantly decrease the FBG level. In glucose tolerance test, AUC was declined in the LQ group compared to the DMC group. As shown in Table 1, QA contained five compounds such as protocatechuic acid (PCA), chlorogenic acid, syringic acid, myricetin, and quercetin. A recent study showed similar tendency that PCA significantly reduced blood glucose and plasma insulin level in the hyperglycemic condition [24]. In addition, it is known that activated ligation of AGEs to renal RAGE activated production of ROS subsequently causing oxidative stress [25]. Furthermore, chlorogenic acid (CGA) and quercetin have been shown to decrease blood glucose level by stimulating glucose uptake through the activation of AMPK in diabetic mice [26,27]. CGA has been also reported as an inhibitor of carbonic anhydrase V which has an impact on gluconeogenesis [28]. The current results showed that a high dose of QAE treatment decreased RAGE expression compared to T2DM mice. These data suggest that the QAE treatment has ameliorative effects on a hyperglycemic condition due to synergistic or additive effects of PCA, chlorogenic acid, and quercetin these active ingredients.

Moreover, there are well-known renal malfunction indicators including albuminuria, plasma creatinine, BUN, and urinary ACR level in DN. Our data showed that the QAE treatment significantly decreased urinary ACR, plasma creatinine, and BUN in diabetic mice. From these changes, it could be inferred that the QAE treatment improved renal function in a diabetic condition.

In terms of molecules, major mechanisms of hyperglycemia-induced tissue damage are as follows—the increase of intracellular AGEs formation and its receptor expression, and activation of PKC. As indicated above, the current study demonstrated that QAE supplementation reduced the protein level of RAGE in the diabetic mice. In addition, elevated protein levels of 4-HNE were decreased in the HQ group and both doses of QAE supplementation lowered protein carbonyls in the DMC group. Previous studies showed that the increased level of Nrf2 as well as 4-HNE and protein carbonyls activated Nrf2 related antioxidant defense systems [12,29]. Our results particularly demonstrated that the protein level of Nrf2 and its related antioxidant defense enzymes including NQO1, HO-1, and catalase were increased in the DMC mice but these markers were reduced in the HQ group. Especially, PCA is known to attenuate oxidative stress by decreasing the levels of ROS and malondialdehyde (MDA) in a diabetic condition [30]. Hence, it can be concluded that QAE containing PCA and CGA supplementation could alleviate cellular oxidative stress as well as activations of RAGE in diabetes.

Oxidative stress also can contribute to inflammatory response via the activation of NF-κB and downstream factors such as TNF-α, IL-6, and iNOS in DN [31]. Furthermore, oxidative stress would potentially activate NLRP3 inflammasome by initial recognition as cellular danger [32,33]. In this study, the renal protein levels of NLRP3 inflammasome, nuclear NF-κB, and subsequent inflammatory factors were higher in the DMC group compared with the NC group. A previous study demonstrated that the PCA treatment significantly reduced the secretion of pro-inflammatory cytokines in T2DM rats [34]. Furthermore, syringic acid is known to reduce oxidative stress and inflammation in diabetes [35]. Simultaneously, QAE supplementation selectively reduced the renal inflammatory factors via suppression of NLRP3 inflammasome. Therefore, the current study suggested that QAE supplementation alleviated the activation of NLRP3 inflammasome and consequential hyper-inflammation under a diabetic condition.

In consistent hyperglycemia, chronic hyper-inflammation in the kidney results in renal apoptosis via activation of caspases, proapoptotic protein Bax, p53, and mitogen activated protein kinase (MAPK) signaling [36,37,38,39]. PCA is known to reduce the protein expression levels of type IV collagen, laminin, and fibronectin in high glucose-stimulated human mesangial cells (MCs) [40]. Our results showed that pro-fibrosis related markers including PKC-βII, TGF-βI, and α-SMA as well as apoptosis related markers such as caspase-8, caspase-3, Bax/Bcl-2 ratio, and pERK/ERK ratio were declined in the QAE treated group, regardless of dose, compared with the DMC group. The present study suggested that PCA and chlorogenic acid in QAE might play major roles in the protection of renal apoptosis and fibrosis in T2DM.

How could the QAE treatment ameliorate the renal damage through suppression of oxidative stress, NLRP3 inflammasome-dependent hyper-inflammation, cell apoptosis, and pro-fibrosis in a hyperglycemic condition? There are cumulative evidences that AMPK influences intracellular signaling pathway, especially amelioration of oxidative stress via activation of antioxidant defense enzymes [41]. Metformin, which is a well-known diabetic drug, shows therapeutic mechanisms related to AMPK, which suppresses the NF-κB through activation of SIRT1 and PGC-1α [21,42,43]. PCA also increased the phosphorylation of AMPK and then activated the expression of p-Nrf2 and HO-1 in oxidative damage in HUVECs [44]. Moreover, a recent study reported that the syringic acid improved energy metabolism by regulation of mitochondrial biogenesis in diabetic rats [33]. On the other hand, SIRT1, an intracellular energy sensor, beneficially affected glucose homeostasis, cellular immunity to oxidative stress, inflammation, apoptosis, and fibrosis in the kidney [45]. In DN, one of the earliest characteristics is the loss of podocyte, which plays a crucial role in albumin processing, but SIRT1 is known to attenuate podocyte depletion and albuminuria by downregulation of claudin-1 in podocytes [46,47]. Resveratrol, a natural plant polyphenol, respectively stimulates SIRT1 and AMPK, and has a protective effect on oxidative stress and inflammatory response in the kidney [20]. A previous study showed that SIRT1 suppressed NLRP3 inflammasome activation as well as the NF-κB associated inflammatory response [48,49]. PGC-1α also regulates oxidative stress via participation in cellular signaling to mitochondrial oxidative stress and independently inhibits the NF-κB related inflammatory response [50,51]. The current studies demonstrated that the treatment of QAE containing PCA and syringic acid elevated the protein levels of SIRT1 and PGC-1α and downregulated NLRP3 inflammasome dependent inflammatory mediators in the diabetic mice. In particular, both doses of QAE supplementation has an effect on the stimulation of AMPK/SIRT pathway and a high dose of QAE supplementation decreased NLRP3 inflammation accompanied by nuclear NF-κB activation in our study. Therefore, it can be inferred that the QAE treatment has a protective effect on renal oxidative stress and hyper-inflammation under a hyperglycemic condition by involving this antagonism of SIRT1/NF-κB/NLRP3 inflammasome.

The previous study reported by our group found that the *Lespedeza bicolor* extract (LBE) containing polyphenolic compounds such as quercetin, genistein, daidzein, and naringenin has shown to exert antioxidant and anti-inflammatory effects accompanied by upregulation of the AMPK-SIRT1 pathway in the same diabetic model [52]. The current findings supported that QAE at much lower concentrations compared to LBE and other plant extracts has shown antidiabetic effects through regulation of the AMPK-SIRT related mechanism as shown in LBE treated diabetic mice [52]. Moreover, QAE supplementation attenuated pro-fibrosis as well as apoptosis in the diabetic group, which was not shown in LBE treatment groups. Therefore, it can be concluded that QAE is more effective than LBE on renal fibrosis and apoptosis in diabetes.

## 5. Conclusions

Taken together, we reported that QAE supplementation at a high dose had ameliorative effects on renal NLRP3 inflammasome associated hyper-inflammation and consequent renal cell apoptosis and pro-fibrosis in the HFD/STZ-induced T2DM mice. In addition, QAE supplementation regardless of the dose stimulated AMPK/SIRT1 signaling and ameliorated oxidative stress, although some molecular markers were selectively regulated at different treatment doses of QAE in diabetic renal damage. In conclusion, the current study suggested that QAE could be a potential therapeutic for improving renal damage in T2DM.

## Figures and Tables

**Table 1:** Contents of protocatechuic acid, chlorogenic acid, syringic acid, myricetin, and quercetin compounds by HPLC analysis in the presence of *Quamoclit angulata* extract (QAE).

Samples	Content (µg/g)				
	Protocatechuic Acid	Chlorogenic Acid	Syringic Acid	Myricetin	Quercetin
QAE	198.86 ± 2.26	54.11 ± 1.81	56.38 ± 0.57	12.42 ± 0.09	13.41 ± 0.08

Data were expressed as means ± SEM (*n* = 3).

**Table 2:** Effect of QAE on body weight, kidney weight, and food intake in type 2 diabetes mellitus (T2DM) mice.

Group	NC	DMC	LQ	HQ
Body Weight (g)				
before treatment	26.58 ± 0.45 ^a^	32.04 ± 1.04 ^b^	31.90 ± 1.25 ^b^	30.80 ± 0.68 ^b^
after treatment	30.42 ± 0.61 ^a^	40.93 ± 1.57 ^b^	40.20 ± 2.20 ^b^	39.40 ± 1.89 ^b^
Gain	3.85 ± 0.33 ^a^	8.89 ± 0.70 ^b^	8.31 ± 1.15 ^b^	8.60 ± 1.33 ^b^
Kidney Weight (mg)	151.00 ± 5.34 ^a^	183.00 ± 14.37 ^b^	175.00 ± 11.18 ^a,b^	176.00 ± 4.30 ^a,b^
Food Intake (g/day)	2.32 ± 0.21 ^a^	3.48 ± 0.25 ^b^	3.3 ± 0.12 ^b^	3.76 ± 0.12 ^b^

Data were expressed as means ± SEM. Mean values with the same superscript letter (a,b) are not significantly different (*p* < 0.05). NC: Normal mice; DMC: Type 2 diabetic mice; LQ: Type 2 diabetic mice supplemented with a low dose (5 mg/kg/day) of QAE; HQ: Type 2 diabetic mice supplemented with a high dose (10 mg/kg/day) of QAE.

**Table 3:** Effect of QAE on plasma indices related to type 2 diabetes.

Plasma Indices	NC	DMC	LQ	HQ
FBG (mg/dL)	122 ± 7.51 ^a^	173 ± 14.30 ^b^	156 ± 9.94 ^a,b^	150 ± 12.04 ^a,b^
Insulin (uU/mL)	1.62 ± 0.30 ^a^	2.62 ± 0.23 ^b^	1.80 ± 0.38 ^a,b^	1.61 ± 0.20 ^a^
HOMA-IR	2.82 ± 0.58 ^a^	8.45 ± 0.79 ^b^	3.80 ± 1.01 ^a^	4.19 ± 0.62 ^a^
HbA1c (%)	6.61 ± 0.51 ^a^	12.18 ± 2.82 ^b^	8.64 ± 0.76 ^a,b^	7.13 ± 0.39 ^a^

Data were expressed as means ± SEM. Mean values with the same superscript letter (a,b) are not significantly different (*p* < 0.05). NC: Normal mice; DMC: Type 2 diabetic mice; LQ: Type 2 diabetic mice supplemented with a low dose (5 mg/kg/day) of QAE; HQ: Type 2 diabetic mice supplemented with a high dose (10 mg/kg/day) of QAE.

## Abbreviations

The following abbreviations are used in this manuscript:

ACR	Albumin/Creatinine ratio
AGEs	Advanced glycation end products
AMPK	5′ adenosine monophosphate-activated protein kinase
ASC	Apoptosis-associated speck-like protein containing a caspase recruitment domain
AUC	Area under the curve
BUN	Blood urea nitrogen
DM	Diabetes mellitus
DN	Diabetic nephropathy
FBG	Fasting blood glucose
GPx	Glutathione peroxidase
4-HNE	4-hydroxynonenal
HO-1	Hemeoxygenase-1
IL-1β	Interleukin-1β
IL-6	Interleukin-6
iNOS	Inducible nitric oxide synthase
NC	Normal control
NF-κB	Nuclear factor-κB
NLRP3	NOD-like pyrin domain containing receptor 3
NQO1	NAD(P)H quinone dehydrogenase-1
PKC	Protein kinase C
RAGE	Receptor for advanced glycation end products
ROS	Reactive oxygen species
SIRT1	Sirtuin1
SMA	Smooth muscle actin
SOD	Superoxide dismutase
T2DM	Type 2 diabetes mellitus
TGF-β	Transforming growth factor-β
TNF-α	Tumor necrosis factor-α
QA	*Quamoclit angulata*
QAE	*Quamoclit angulata* extract

## References

[1] Rains J.L., Jain S.K. (2011). Oxidative stress, insulin signaling, and diabetes. Free Radic. Biol. Med..

[2] Wright E., Scism-Bacon J.L., Glass L.C. (2006). Oxidative stress in type 2 diabetes: The role of fasting and postprandial glycaemia. Int. J. Clin. Pract..

[3] Tesch G.H., Allen T.J. (2007). Rodent models of streptozotocin-induced diabetic nephropathy. Nephrology.

[4] Ritz E. (2006). Diabetic nephropathy. Saudi J. Kidney Dis. Transpl..

[5] Turkmen K. (2017). Inflammation, oxidative stress, apoptosis, and autophagy in diabetes mellitus and diabetic kidney disease: The Four Horsemen of the Apocalypse. Int. Urol. Nephrol..

[6] Eo H., Lee H.J., Lim Y. (2016). Ameliorative effect of dietary genistein on diabetes induced hyper-inflammation and oxidative stress during early stage of wound healing in alloxan induced diabetic mice. Biochem. Biophys. Res. Commun..

[7] Newsholme P. (2016). Molecular mechanisms of ROS production and oxidative stress in diabetes. Biochem. J..

[8] Habib S.L. (2013). Diabetes and renal tubular cell apoptosis. World J. Diabetes.

[9] Noh H., Ha H. (2011). Reactive oxygen species and oxidative stress. Contrib. Nephro..

[10] Lu M. (2017). Curcumin Ameliorates Diabetic Nephropathy by Suppressing NLRP3 Inflammasome Signaling. Biomed. Res. Int..

[11] He Y., Hara H., Nunez G. (2016). Mechanism and Regulation of NLRP3 Inflammasome Activation. Trends Biochem. Sci..

[12] Eo H., Park J.E., Jeon Y.J., Lim Y. (2017). Ameliorative Effect of Ecklonia cava Polyphenol Extract on Renal Inflammation Associated with Aberrant Energy Metabolism and Oxidative Stress in High Fat Diet-Induced Obese Mice. J. Agric. Food Chem..

[13] Shen J., Wang L., Jiang N., Mou S., Zhang M., Gu L., Shao X., Wang Q., Qi C., Li S. (2016). NLRP3 inflammasome mediates contrast media-induced acute kidney injury by regulating cell apoptosis. Sci. Rep..

[14] Klen J., Goricar K., Janez A., Dolzan V. (2015). NLRP3 Inflammasome Polymorphism and Macrovascular Complications in Type 2 Diabetes Patients. J. Diabetes Res..

[15] Wagener F.A., Dekker D., Berden J.H., Scharstuhl A., van der Vlag J. (2009). The role of reactive oxygen species in apoptosis of the diabetic kidney. Apoptosis.

[16] Balasescu E., Ion D.A., Cioplea M., Zurac S. (2015). Caspases, Cell Death and Diabetic Nephropathy. Rom. J. Intern. Med..

[17] Dakshinamurty K.V. (2013). Pathophysiology and pathology of Diabetic Nephropathy. Diabetic Kidney Disease—ECAB.

[18] Chen S., Hong S.W., Iglesias-de la Cruz M.C., Isono M., Casaretto A., Ziyadeh F.N. (2001). The key role of the transforming growth factor-beta system in the pathogenesis of diabetic nephropathy. Ren. Fail..

[19] Kitada M., Koya D. (2013). SIRT1 in Type 2 Diabetes: Mechanisms and Therapeutic Potential. Diabetes Metab. J..

[20] Kim Y., Park C.W. (2016). Adenosine monophosphate-activated protein kinase in diabetic nephropathy. Kidney Res. Clin. Pract..

[21] Chun B.H. (2011). Pharmaceutical Composition for Treating Diabetes Containing Quamoclit Angulate Extracts. U.S. Patent.

[22] Zhang M., Lv X.Y., Li J., Xu Z.G., Chen L. (2008). The characterization of high-fat diet and multiple low-dose streptozotocin induced type 2 diabetes rat model. Exp. Diabetes Res..

[23] Han B.G., Hao C.M., Tchekneva E.E., Wang Y.Y., Lee C.A., Ebrahim B., Harris R.C., Kern T.S., Wasserman D.H., Breyer M.D. (2008). Markers of glycemic control in the mouse: Comparisons of 6-h- and overnight-fasted blood glucoses to Hb A1c. Am. J. Physiol. Endocrinol. Metab..

[24] Semaming Y., Kukongviriyapan U., Kongyingyoes B., Thukhammee W., Pannangpetch P. (2016). Protocatechuic Acid Restores Vascular Responses in Rats With Chronic Diabetes Induced by Streptozotocin. Phytother. Res..

[25] Sourris K.C., Morley A.L., Koitka A., Samuel P., Coughlan M.T., Penfold S.A., Thomas M.C., Bierhaus A., Nawroth P.P., Yamamoto H. (2010). Receptor for AGEs (RAGE) blockade may exert its renoprotective effects in patients with diabetic nephropathy via induction of the angiotensin II type 2 (AT2) receptor. Diabetologia.

[26] Ong K.W., Hsu A., Tan B.K. (2012). Chlorogenic acid stimulates glucose transport in skeletal muscle via AMPK activation: A contributor to the beneficial effects of coffee on diabetes. PLoS ONE.

[27] Dhanya R., Arya A.D., Nisha P., Jayamurthy P. (2017). Quercetin, a Lead Compound against Type 2 Diabetes Ameliorates Glucose Uptake via AMPK Pathway in Skeletal Muscle Cell Line. Front. Pharmacol..

[28] Mollica A., Locatelli M., Macedonio G., Carradori S., Sobolev A.P., De Salvador R.F., Monti S.M., Buonanno M., Zengin G., Angeli A. (2016). Microwave-assisted extraction, HPLC analysis, and inhibitory effects on carbonic anhydrase I, II, VA, and VII isoforms of 14 blueberry Italian cultivars. J. Enzyme Inhib. Med. Chem..

[29] Izumi Y., Yamamoto N., Matsushima S., Yamamoto T., Takada-Takatori Y., Akaike A., Kume T. (2015). Compensatory role of the Nrf2-ARE pathway against paraquat toxicity: Relevance of 26S proteasome activity. J. Pharmacol. Sci..

[30] Adedara I.A., Fasina O.B., Ayeni M.F., Ajayi O.M., Farombi E.O. (2019). Protocatechuic acid ameliorates neurobehavioral deficits via suppression of oxidative damage, inflammation, caspase-3 and acetylcholinesterase activities in diabetic rats. Food Chem. Toxicol..

[31] Salminen A., Hyttinen J.M., Kaarniranta K. (2011). AMP-activated protein kinase inhibits NF-kappaB signaling and inflammation: Impact on healthspan and lifespan. J. Mol. Med..

[32] Harijith A., Ebenezer D.L., Natarajan V. (2014). Reactive oxygen species at the crossroads of inflammasome and inflammation. Front. Physiol..

[33] Salminen A., Kaarniranta K., Kauppinen A. (2013). Crosstalk between Oxidative Stress and SIRT1: Impact on the Aging Process. Int. J. Mol. Sci..

[34] Bhattacharjee N., Dua T.K., Khanra R. (2017). Protocatechuic Acid, a Phenolic from Sansevieria roxburghiana Leaves, Suppresses Diabetic Cardiomyopathy via Stimulating Glucose Metabolism, Ameliorating Oxidative Stress, and Inhibiting Inflammation. Front. Pharmacol..

[35] Shariati H., Hassanpour M., Sharifzadeh G., Zarban A., Samarghandian S., Saeedi F. (2020). Evaluation of diuretic and antioxidant properties in aqueous bark and fruit extracts of pine. Curr. Drug Discov. Technol..

[36] Elmore S. (2007). Apoptosis: A review of programmed cell death. Toxicol. Pathol..

[37] Towns R., Pietropaolo M., Wiley J.W. (2008). Stimulation of autophagy by autoantibody-mediated activation of death receptor cascades. Autophagy.

[38] Giacco F., Brownlee M. (2010). Oxidative stress and diabetic complications. Circ. Res..

[39] Fu J., Lee K., Chuang P.Y., Liu Z., He J.C. (2015). Glomerular endothelial cell injury and cross talk in diabetic kidney disease. Am. J. Physiol. Renal Physiol..

[40] Ma Y., Chen F., Yang S., Chen B., Shi J. (2018). Protocatechuic acid ameliorates high glucose-induced extracellular matrix accumulation in diabetic nephropathy. Biomed. Pharmacother..

[41] Wang K., Tang Z., Wang J., Cao P., Li Q., Shui W., Wang H., Zheng Z., Zhang Y. (2017). RETRACTED: Polysaccharide from Angelica sinensis ameliorates high-fat diet and STZ-induced hepatic oxidative stress and inflammation in diabetic mice by activating the Sirt1-AMPK pathway. J. Nutr. Biochem..

[42] Yacoub R., Lee K., He J.C. (2014). The Role of SIRT1 in Diabetic Kidney Disease. Front. Endocrinol..

[43] Wan X., Wen J.J., Koo S.J., Liang L.Y., Garg N.J. (2016). SIRT1-PGC1alpha-NFkappaB Pathway of Oxidative and Inflammatory Stress during Trypanosoma cruzi Infection: Benefits of SIRT1-Targeted Therapy in Improving Heart Function in Chagas Disease. PLoS Pathog..

[44] Han L., Yang Q., Ma W., Li J., Qu L., Wang M. (2018). Protocatechuic Acid Ameliorated Palmitic-Acid-Induced Oxidative Damage in Endothelial Cells through Activating Endogenous Antioxidant Enzymes via an Adenosine-Monophosphate-Activated-Protein-Kinase-Dependent Pathway. J. Agric. Food Chem..

[45] Guclu A., Erdur F.M., Turkmen K. (2016). The Emerging Role of Sirtuin 1 in Cellular Metabolism, Diabetes Mellitus, Diabetic Kidney Disease and Hypertension. Exp. Clin. Endocrinol. Diabetes.

[46] Hasegawa K., Wakino S., Simic P., Sakamaki Y., Minakuchi H., Fujimura K., Hosoya K., Komatsu M., Kaneko Y., Kanda T. (2013). Renal tubular Sirt1 attenuates diabetic albuminuria by epigenetically suppressing Claudin-1 overexpression in podocytes. Nat. Med..

[47] Kong L., Wu H., Zhou W., Luo M., Tan Y., Miao L., Cai L. (2015). Sirtuin 1: A Target for Kidney Diseases. Mol. Med..

[48] Lee H.J., Hong Y.S., Yang S.J. (2015). Interaction between NLRP3 Inflammasome and Sirt1/6: Metabolomics Approach. FASEB J..

[49] Li Y., Yang X., He Y., Wang W., Zhang J., Zhang W., Jing T., Wang B., Lin R. (2017). Negative regulation of NLRP3 inflammasome by SIRT1 in vascular endothelial cells. Immunobiology.

[50] Guo K., Lu J., Huang Y., Wu M., Zhang L., Yu H., Zhang M., Bao Y., He J.C., Chen H. (2015). Protective role of PGC-1alpha in diabetic nephropathy is associated with the inhibition of ROS through mitochondrial dynamic remodeling. PLoS ONE.

[51] Liang H., Ward W.F. (2006). PGC-1alpha: A key regulator of energy metabolism. Adv. Physiol. Educ..

[52] Park J.E., Lee H., Kim S.Y., Lim Y. (2020). *Lespedeza bicolor* Extract Ameliorated Renal Inflammation by Regulation of NLRP3 Inflammasome-Associated Hyperinflammation in Type 2 Diabetic Mice. Antioxidants.

